# ^68^Ga-PSMA PET/CT: effect of external cooling on salivary gland uptake

**DOI:** 10.1590/0100-3984.2020.0044

**Published:** 2021

**Authors:** Matheus Zanelatto Junqueira, Nelisa Helena Rocha, Marcelo Tatit Sapienza

**Affiliations:** 1 Faculdade de Medicina da Universidade de São Paulo (FMUSP), São Paulo, SP, Brazil.; 2 Instituto do Câncer do Estado de São Paulo Octavio Frias de Oliveira (Icesp), São Paulo, SP, Brazil.

**Keywords:** Salivary glands, Hypothermia, induced, Positron emission tomography computed tomography, ^68^Ga-PSMA, ^177^Lu-PSMA-617, Prostatic neoplasms, castration-resistant, Glândulas salivares, Hipotermia induzida, Tomografia computadorizada com tomografia por emissão de pósitrons, ^68^Ga-PSMA, ^177^Lu-PSMA-617, Neoplasias da próstata resistentes à castração

## Abstract

**Objective:**

To evaluate the effect that external cooling of the salivary glands (ECSG) has on the uptake of gallium-68-labeled prostate-specific membrane antigen (^68^Ga-PSMA), as an indirect assessment of the capacity of ECSG to reduce the local dose in lutetium-177-PSMA-617 radioligand therapy.

**Materials and Methods:**

Ten patients with prostate cancer were submitted to ^68^Ga-PSMA positron emission tomography/computed tomography with unilateral ECSG. The ECSG was started at 30 min before the injection of the radiotracer and maintained until the end of image acquisition (1 h after injection). Each salivary gland was assessed by determining the maximum, mean, and peak standardized uptake values (SUVmax, SUVmean, and SUVpeak, respectively). The volume of each gland was determined in a volume of interest delineated by a threshold SUVmax of 10%. Paired Student’s t-tests were used in order to compare the results.

**Results:**

In terms of the SUV parameters, there were no statistically significant differences between the cooled and contralateral salivary glands. However, the mean volume was 27% lower in the cooled parotid glands than in the contralateral parotid glands (*p* = 0.004).

**Conclusion:**

The use of ECSG does not appear to reduce ^68^Ga-PSMA uptake by the salivary glands. In addition, there is yet no evidence that ECSG is effective in preventing salivary gland toxicity.

## Abstract (pt)

**Objetivo:**

Avaliar o impacto do resfriamento externo de glândulas salivares (REGS) na captação de ^68^Ga-PSMA como marcador indireto dessa intervenção para redução da dose local na terapia com ^177^Lu-PSMA.

**Materiais e Métodos:**

Dez pacientes com câncer de próstata foram submetidos a PET/CT com ^68^Ga-PSMA com REGS unilateral. O resfriamento se iniciou 30 minutos antes da injeção do radiofármaco até o fim da aquisição de imagem, 1 hora após a injeção. Cada glândula foi avaliada para os valores de captação padronizados máximo, médio e pico (SUVmáx, SUVmédio e SUVpico, respectivamente). O volume foi definido por um isocontorno usando 10% do SUVmáx. Os resultados foram comparados com o teste *t* de Student.

**Resultados:**

Não houve diferença estatisticamente significante entre os valores de SUV das glândulas resfriadas e seus controles. Houve 27% de redução volumétrica (*p* = 0,004) nas parótidas resfriadas em comparação ao controle.

**Conclusão:**

Não houve redução da captação de ^68^Ga-PSMA nas glândulas salivares ao REGS. Atualmente não há evidências que suportem essa prática clínica.

## INTRODUCTION

Prostate cancer is one of the most prevalent types of cancer among men in Brazil and worldwide, second only to nonmelanoma skin cancer, and accounts for up to a quarter of newly diagnosed cases of cancer^(1,2)^. In Brazil, there were 15,391 reported deaths from prostate cancer in 2018^(3)^, and it is estimated that there will be more than 65,000 new cases in 2020. In recent years, radiolabeled prostate-specific membrane antigen (PSMA) ligands have emerged as promising alternatives in the management of prostate cancer, following the modern trend of precision medicine^(4,5)^. Positron emission tomography/computed tomography (PET/CT) employing PSMA ligands radiolabeled with positron emitters such as gallium-68 (^68^Ga) is used in the restaging of patients with biochemical recurrence and in other clinical scenarios^(4)^.

Lutetium-177 (^177^Lu)-labeled radiopharmaceuticals are being investigated as therapeutic alternatives in castrate-resistant prostate cancer, with good prospects^(5,6)^. However, one of the major adverse effects of this therapy is the salivary gland toxicity associated with the uptake of PSMA-targeted radiopharmaceuticals, leading to xerostomia, halitosis, and poor quality of life^(7,8)^. The mechanism of that uptake has yet to be elucidated, and new evidence points to a nonspecific mechanism, different from that responsible for the antitumor activity of such radiopharmaceuticals and probably independent of the level of PSMA expression in the plasma membrane^(9)^. Therefore, techniques to reduce salivary gland toxicity constitute a topic of interest for groups involved in research and in the clinical use of PSMA therapy^(7)^. One such technique is external cooling of the salivary glands (ECSG) with ice packs, similar to the scalp cooling applied for the prevention of alopecia during some chemotherapy regimens. The rationale is that cooling will reduce blood flow by eliciting a peripheral vasoconstriction response, consequently reducing the amount of radiopharmaceutical and radiation delivered to the gland. Despite having been used empirically in European protocols, there is little evidence that this technique is effective^(10)^. Although ^68^Ga- and ^177^Lu-labeled PSMA ligands do not have the same exact pharmacological and nuclear characteristics, ^68^Ga has been used as a marker of ^177^Lu uptake in the evaluation of salivary glands, due to its lower cost and greater safety^(7,10)^.

The aim of this study was to evaluate the effect of ECSG on the uptake of ^68^Ga PSMA by the salivary glands, as an indirect assessment of the ability of this intervention to reduce ^177^Lu PSMA-617 therapy-related salivary gland toxicity.

## MATERIALS AND METHODS

In this study, we evaluated the effect of unilateral cooling of the parotid and submandibular salivary glands in ten patients undergoing ^68^Ga-PSMA PET/CT. The PET/CT examination followed the standard protocol adopted by our department of nuclear medicine, with minor modifications, as described below. Patients had been referred, from the departments of urology and oncology, for staging/restaging. The study was approved by the local research ethics committee (Reference no. 2.897.862), and all participating patients gave written informed consent.

**Figure 1:**
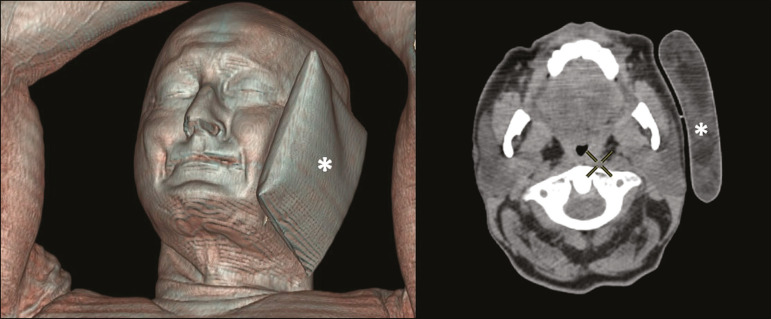
Image showing the use of the external cooling device (ice pack) on the left side (asterisk).

Cooling was performed by attaching a neoprene support containing an ice pack to one side of the face of the patient, in close contact with the parotid gland (Figure 1). Contact with the submandibular gland was hampered by its anatomical position and the curvature of the jaw. Cooling started 30 min before the injection of 191.7 MBq (5.2 ± 0.9 mCi) of the radiotracer. Ice packs were replaced every 30 min, and their temperatures were measured and recorded using infrared thermometers, to monitor the temperature standardization.

**Figure 2:**
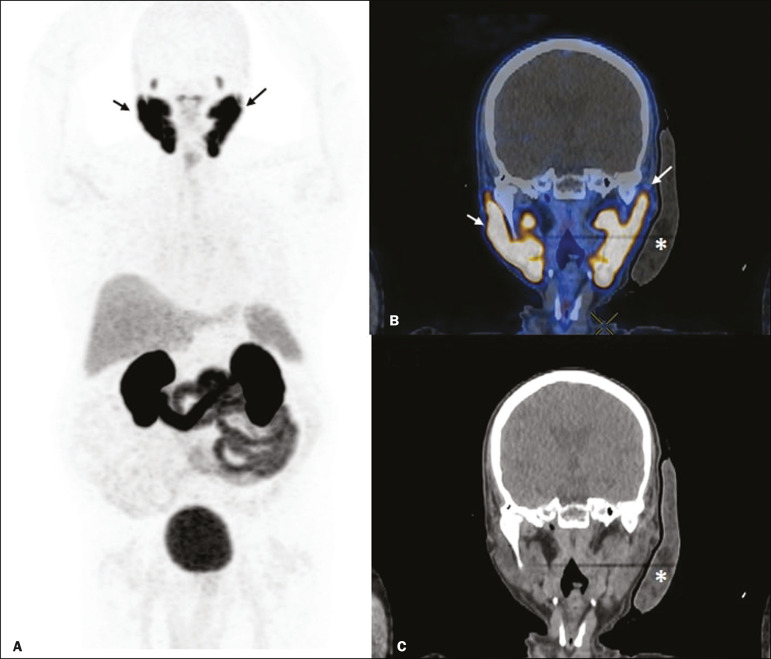
Maximum intensity projection PET (**A**) and coronal fused PET/CT (**B**) demonstrating physiologically accentuated uptake of 68Ga-PSMA by salivary glands (arrows) at t1. Coronal fused PET/CT (**B**) and CT (**C**) showing the position of the external cooling device (asterisks).

Image acquisition started at 1 h after injection of ^68^Ga-PSMA, designated time point 1 (t1), and continued for approximately 30 min (Figure 2). Cooling was stopped shortly after the t1 acquisition. A head and neck acquisition at 4 h after injection, designated time point 4 (t4), was the only other modification of the standard protocol during the experiment. Using the OsiriX Dicom Viewer Lite free software (Pixmeo, Geneva, Switzerland), we quantitatively evaluated the maximum, mean, and peak standardized uptake value (SUVmax, SUVmean, and SUVpeak, respectively) for the cooled and contralateral (control) parotid glands, as well as for the cooled and contralateral (control) submandibular glands. The SUVpeak was measured in a 1 cm^3^ volume around the point of SUVmax. The fourth parameter was volume, assessed for a volume of interest delineated by a threshold SUVmax of 10%. The glandular volume and SUV parameters were compared by using paired Student’s *t*-tests and were analyzed with the R Commander software, version 2.6-0 (http://socserv.mcmaster.ca/jfox/Misc/Rcmdr/).

Given the limited knowledge of the physiological uptake of PSMA by the salivary glands, we performed an additional, retrospective, analysis of the PET/CT scans of seven patients not submitted to cooling. That analysis was also approved by the local research ethics committee (Reference no. 3.827.054). Images were also processed with the R Commander software, the SUVmax, SUVmean, and SUVpeak, as well as the parotid volumes, having been obtained in a t1 acquisition. Because those PET/CT scans were performed with the standard protocol, t4 acquisitions were not available.

In all 17 patients, the anteroposterior and laterolateral diameters were measured on the basis of the CT of the parotid gland, in order to obtain anatomical confirmation of the changes in volume observed in the functional PET study. The diameters were measured manually, with a window of −30 HU to 50 HU. Data for patients with and without cooling were compared by using unpaired Student’s *t*-test, with the aid of the R Commander software.

## RESULTS

**Figure 3:**
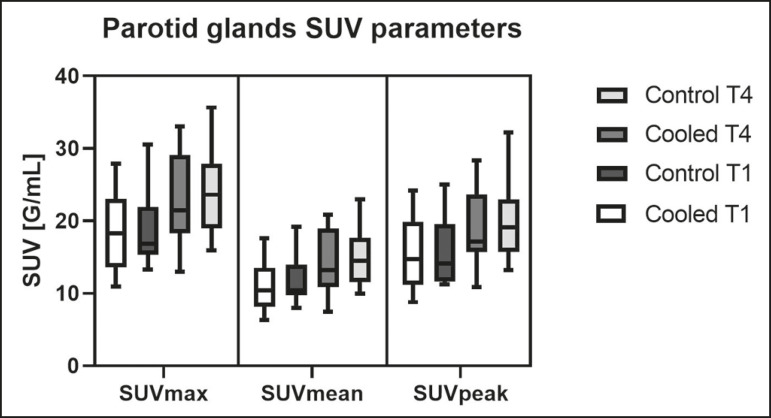
Box plot showing SUV parameters for cooled and contralateral parotid glands, at t1 and t4.

**Figure 4:**
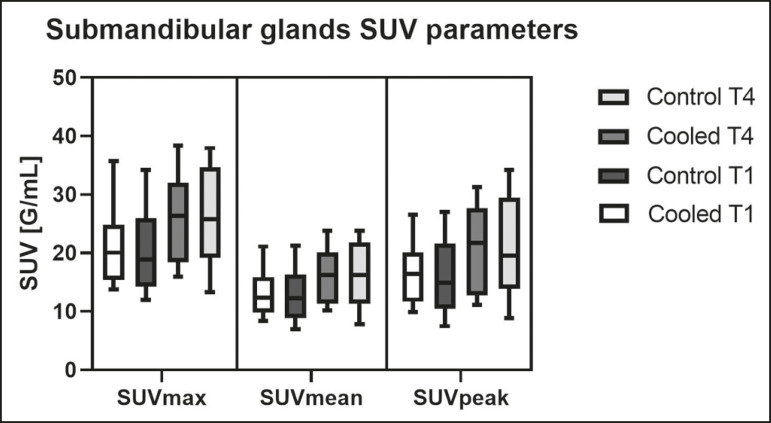
Box plot showing SUV parameters for cooled and contralateral submandibular glands, at t1 and t4.

All of the patients evaluated had castrate-resistant prostate cancer. The mean age was 69.2 ± 5.7 years. Comparing the cooled glands with the contralateral glands, we found no statistically significant difference in any of the SUV parameters at t1 or t4, despite finding that those parameters were 3-8% and 4-7% lower at t1 and t4, respectively, in the cooled parotid glands. In the cooled submandibular glands, all of the SUV parameters actually increased by 0-3%, although that difference was also not statistically significant. The mean parotid gland volume was lower in the cooled glands than in the contralateral glands, being significantly lower at t1 (22.0 cm^3^ vs. 28.0 cm^3^, a difference of 27.2%; *p* = 0.004), although not at t4 (25.3 cm^3^ vs. 28.1 cm^3^, a difference of 9.8%; *p* = 0.07). Data for the parotid and submandibular glands are summarized in box plots in Figures 3 and 4, respectively. For all of the glands evaluated in the prospective analysis, the t1 and t4 data are detailed in Tables 1 and 2, respectively.

**Table 1:** Uptake of ^68^Ga PSMA by and volume of cooled and contralateral sali- vary glands at t1.

Variable	Cooled (n = 10) Mean ± SD	Contralateral (n = 10) Mean ± SD	Variation (%)	*P*
Parotid glands				
SUVmax	18.4 ± 6.0	18.9 ± 5.5	-2.9	0.56
SUVpeak	15.3 ± 5.3	15.9 ± 4.8	-3.9	0.49
SUVmean	11.0 ± 3.7	11.9 ± 3.6	-8.1	0.12
Volume (cm^3^)	22.0 ± 7.1	28.0 ± 8.2	-27.2	0.004
Submandibular glands				
SUVmax	20.9 ± 6.8	20.3 ± 7.4	2.7	0.39
SUVpeak	16.4 ± 5.4	16.0 ± 6.6	2.7	0.61
SUVmean	13.1 ± 4.1	12.9 ± 4.6	2.0	0.59
Volume (cm^3^)	9.1 ± 2.1	8.8 ± 2.9	3.4	0.67

**Table 2:** Uptake of ^68^Ga PSMA by and volume of cooled and contralateral sali- vary glands at t4.

Variable	Cooled (n = 10) Mean ± SD	Contralateral (n = 10) Mean ± SD	Variation (%)	*P*
Parotid glands				
SUVmax	22.8 ± 6.5	23.6 ± 6.0	-4.1	0.37
SUVpeak	19.0 ± 5.6	20.0 ± 5.8	-6.4	0.24
SUVmean	14.0 ± 4.4	14.8 ± 4.1	-7.0	0.18
Volume (cm^3^)	25.3 ± 6.3	28.1 ± 6.8	-9.8	0.07
Submandibular glands				
SUVmax	26.2 ± 7.8	26.1 ± 8.6	0.3	0.92
SUVpeak	21.1 ± 7.5	20.6 ± 8.6	2.6	0.54
SUVmean	16.2 ± 4.9	16.2 ± 5.7	0.3	0.91
Volume (cm^3^)	9.0 ± 2.1	8.3 ± 2.6	7.9	0.06

Table 3 shows the comparison between the prospective and retrospective samples of parotid glands, in terms of the SUV parameters and volumes. There were no significant differences between the SUV parameters obtained for the cooled parotid glands and those obtained for the parotid glands in the seven patients not submitted to cooling. However, the mean parotid gland volume at t1 was significantly lower in the cooled parotid glands than in those evaluated retrospectively (22.0 cm^3^ vs. 30.0 cm^3^, a difference of 37.0%; *p* = 0.01). In terms of the laterolateral and anteroposterior gland diameters, we found no significant differences between the cooled and contralateral glands evaluated in the prospective analysis or between the glands evaluated in the prospective analysis and those evaluated in the retrospective analysis.

**Table 3:** Uptake of ^68^Ga PSMA by and volume of cooled parotid glands (pro- spective analysis) and parotid glands not submitted to cooling (retrospective analysis).

Variable	Cooled (unilateral) (n = 10*) Mean ± SD	Not cooled (bilateral) (n = 14*) Mean ± SD	Variation (%)	*P*
Parotid glands				
SUVmax	18.4 ± 6.0	19.5 ± 8.2	-6	0.73
SUVpeak	15.3 ± 5.3	15.6 ± 6.3	-2	0.91
SUVmean	11.0 ± 3.7	12.3 ± 5.0	-11	0.51
Volume (cm^3^)	22.0 ± 7.1	30.0 ± 7.1	-37	0.01

* Ten patients (10 parotid glands) were evaluated in the prospective analysis, and 7 patients (14 parotid glands) were evaluated in the prospective analysis.

## DISCUSSION

Among the ten patients evaluated prospectively, there were no significant differences between the cooled glands and the contralateral (control) glands, in terms of the SUV parameters. Two previous studies reported the use of ECSG to prevent the uptake of PSMA ligands by salivary glands, both of which were published after the beginning of our investigation. Van Kalmthout et al.^(10)^ used ^68^Ga-PSMA PET/CT to measure salivary gland uptake. The authors showed that SUVmax and SUVpeak were 10-15% lower in the cooled glands of an intervention group (n = 44; cooled bilaterally and unilaterally in 20 and 24, respectively) than in those of a control group (n = 45). It is not clear if such a reduction in uptake would translate to a proportional reduction in tissue damage. In a study of 19 patients evaluated with ^177^Lu-PSMA-617 single-photon emission CT, Yilmaz et al.^(11)^ found no difference between cooled and contralateral (control) salivary glands, thus corroborating our findings. Assuming that ^177^Lu-PSMA-617 uptake is proportional to that of ^68^Ga-PSMA, our data suggest that ECSG is not effective in reducing the radiation dose and salivary gland toxicity during radionuclide therapy.

Albeit intuitive, our initial rationale may be wrong, either because we assume that the uptake of PSMA ligands has a direct correlation with the perfusion and that the vascularization of the salivary glands would respond to the cold with significant vasoconstriction. In fact, we found no direct studies of the vascular response of salivary glands to cold. However, the physiological functioning of the carotid artery is better understood: it dilates with cold and contracts with heat^(12-15)^. The salivary glands are irrigated by branches of the common carotid artery and could therefore present a comparable physiological response to temperature, or the proximity of the ice pack to the carotid artery could affect the perfusion of the glands.

It is of note that the initial planning of the study included an arm with cooling for the entire period of the acquisition protocol (until the end of t4). However, the first patient submitted to the experiment presented facial erythema, developing first-degree frostbite. Therefore, we decided to exclude the arm with prolonged cooling, in order to ensure the safety of the patients. It should be borne in mind that, even if presumably safe, external cooling is uncomfortable for the patient and is not without risks.

Our study has some limitations. The experiment was initially designed to include a sample of 30 patients, with a planned partial analysis to be performed after the tenth patient. Considering the findings obtained at t1 and t4 for the first ten patients, together with the high cost of the procedure, it was decided that the experiment should be discontinued at that point. That reduction in the sample size could have limited the statistical power to a degree that precluded the identification of variations of lesser magnitude. Although the inclusion of a larger number of patients could have modified the results of the statistical analysis, even a significant decrease in the SUVs of the cooled glands would have a small effect size and would probably not lead to modification of the radionuclide therapy protocol. Our results show that, overall, the cooled salivary glands and their corresponding controls did not differ significantly in terms of the SUV parameters. However, the mean volume of the cooled parotid glands was significantly (27%) lower than was that of the contralateral parotid glands, as corroborated by the fact that the mean volume of the cooled parotid glands was also significantly lower than was that of the parotid glands evaluated retrospectively. To our knowledge, this is the first time that this finding has been described.

Because the t1 images were acquired during ECSG, one possible explanation for the volume reduction in the parotid glands is extrinsic mechanical compression of the glands secondary to the cooling device. Although the local pressure imposed by ECSG was not measured, we do not believe that the device was tight enough to result in significant tissue compression. The laterolateral and anteroposterior diameters were measured in order to determine whether there was compression or distortion of the gland. The absence of any statistically significant differences in the laterolateral or anteroposterior diameter belies the explanation of extrinsic compression. However, there is a major limitation to the anatomical delimitation of the parotid gland on CT, because fatty infiltration makes its density comparable to that of the adjacent tissues. Similarly, we choose to use a 10% SUVmax threshold to determine the gland volume, an approach that is to some degree prone to inaccuracies because the volume becomes a variable that is assessed indirectly. Nevertheless, the paired approach of our study design was expected to minimize that problem. The authors of the other previously mentioned studies also found no other way to overcome this limitation and resorted to the SUVmax threshold method^(10,11)^.

Another explanation for the volume reduction would be that cooling had a direct impact on the gland, causing it to contract. It is known that the parotid gland is relatively rich in myoepithelial cells^(16)^, which gives it a certain capacity for contraction, and studies of the nasal mucosa have shown that the extracellular matrix alone has the capacity to contract in response to cold^(17)^. The same mechanisms could be postulated to explain the observed reduction in parotid gland volume.

## CONCLUSIONS

This study provides no indication that ECSG reduces the uptake of ^68^Ga-PSMA, and there is no evidence that this technique, which is not completely free of risk, provides protection against salivary gland toxicity. Ideally, this question would be answered by assessing dosimetry and clinical outcomes after ECSG or other interventions in patients treated with ^177^Lu-PSMA-617 ligands. Cooling appears to have a transient effect of reducing the volume of the parotid gland, possibly by triggering a contractile response in the glandular tissue or its stroma.
